# Translating Gastric Cancer Genomics into Targeted Therapy: Mechanistic Insights from Animal Models and Patient-Derived Systems

**DOI:** 10.3390/cells15040365

**Published:** 2026-02-18

**Authors:** Rong-Yaun Shyu, Lu-Kai Wang, Fu-Ming Tsai

**Affiliations:** 1 Department of Internal Medicine, Taipei Tzu Chi Hospital, Buddhist Tzu Chi Medical Foundation, New Taipei City 231, Taiwan; ryshyu866@gmail.com; 2 National Center for Biomodels, National Institutes of Applied Research, Taipei 115, Taiwan; 2407026@niar.org.tw; 3 Department of Research, Taipei Tzu Chi Hospital, Buddhist Tzu Chi Medical Foundation, New Taipei City 231, Taiwan

**Keywords:** gastric cancer, oncogenic dependency, translational research, patient-derived organoids, CLDN18.2, HER2

## Abstract

**Highlights:**

**What are the main findings?**

- Many gastric cancer-targeted therapies that showed efficacy in animal models (e.g., EGFR, MET, FGFR2, and PI3K inhibition) failed to improve survival in clinical trials, revealing major translational limitations.
- HER2- and CLDN18.2-targeted therapies represent rare successful examples of bench-to-bedside translation, supported by clear oncogenic dependency and precise biomarker-driven patient selection.

**What are the implications of the main findings?**

- True tumor dependency and functional biomarker accuracy are critical determinants of successful clinical translation in gastric cancer.
- Advanced patient-relevant models, including PDX, organoids, and organ-on-a-chip platforms, may improve target validation and reduce preclinical–clinical discrepancies.

**Abstract:**

Gastric cancer remains a leading cause of cancer-related mortality worldwide and is marked by pronounced molecular heterogeneity. Advances in genomic profiling have identified key genetic alterations, including oncogenes (*HER2*, *PIK3CA*, and *MYC*), tumor suppressor genes (*TP53*, *CDH1*, and *ARID1A*), and regulators of genome stability and cell architecture (*MLH1*, *RHOA*, and *CLDN18*), which have driven the development of targeted therapeutic strategies. Although genetically engineered mouse models and xenograft systems have been indispensable for functional validation and preclinical drug testing, many approaches that showed promising efficacy in animal models—such as inhibition of EGFR, MET, FGFR2, and the PI3K pathway—failed to translate into overall survival benefits in clinical trials, highlighting major translational limitations. In contrast, HER2- and CLDN18.2-targeted therapies represent rare but notable clinical successes, underscoring the importance of true oncogenic dependency, precise biomarker-driven patient selection, and robust preclinical validation. In this review, we systematically categorize gastric cancer-associated genes according to their biological functions, summarize representative animal models, and critically examine key successes and failures in clinical translation, emphasizing the need for biologically faithful models and precision-driven translational strategies.

## 1. Introduction

Gastric cancer remains one of the leading causes of cancer-related mortality worldwide and is characterized by pronounced molecular heterogeneity and an unfavorable prognosis [1]. With advances in high-throughput sequencing technologies, numerous genetic alterations closely associated with gastric carcinogenesis and disease progression have been identified. These include oncogenes such as *HER2* (*ERBB2*), *PIK3CA*, and *MYC*; tumor suppressor genes such as *TP53*, *adenomatous polyposis coli* (*APC*), *E-cadherin* (*CDH1*), and *ARID1A*; and genes involved in genomic stability and cellular architecture, including *MLH1*, *RHOA*, and *CLDN18*. Collectively, these discoveries have substantially deepened our understanding of the molecular mechanisms underlying gastric cancer and have facilitated the development of targeted and immunotherapeutic strategies, gradually ushering gastric cancer management into the era of precision medicine [2].

**Figure 1:**
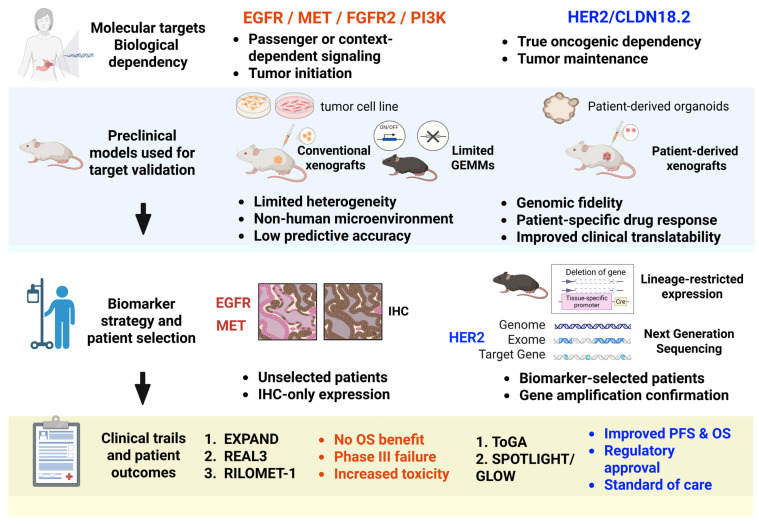
Conceptual framework illustrating determinants of successful versus failed translational targeting in gastric cancer. Schematic comparison of successful (right) and unsuccessful (left) bench-to-bedside translation pathways. Targeted therapies against EGFR, MET, FGFR2, and PI3K showed efficacy in conventional animal models but failed in clinical trials due to passenger signaling, compensatory pathways, and inadequate biomarker selection. In contrast, HER2 and CLDN18.2 represent true tumor maintenance dependencies validated using patient-relevant models, enabling successful clinical translation and regulatory approval. This figure was created in BioRender.

In the course of anticancer drug development, animal models—particularly genetically engineered mouse models (GEMMs) and xenograft systems—have long been employed to validate oncogenic functions and evaluate therapeutic responses [3,4]. However, accumulating clinical evidence indicates that several targeted therapies demonstrating promising efficacy in preclinical animal studies, such as inhibitors targeting epidermal growth factor receptor (EGFR), MET, fibroblast growth factor receptor 2 (FGFR2), or PI3K, have failed to improve overall survival (OS) in large-scale clinical trials. These discrepancies underscore the limitations of conventional animal models in faithfully recapitulating the biological behavior of human gastric cancer. This translational gap—often described as “preclinical success but clinical failure”—can be partly attributed to the marked intra- and intertumoral heterogeneity of gastric cancer, as well as the inability of animal models to fully mimic the human tumor microenvironment and immune interactions. To conceptually illustrate the fundamental differences between successful and unsuccessful translational strategies in gastric cancer, Figure 1 summarizes key determinants of therapeutic efficacy, including true oncogenic dependency, relevance to tumor maintenance rather than tumor initiation, and the distinction between driver and passenger or context-dependent signaling. This framework provides a unifying perspective for understanding why certain targets, such as HER2 and Claudin-18.2 (CLDN18.2), achieved clinical success, whereas others failed despite promising preclinical results.

In recent years, patient-derived models have emerged as pivotal tools for bridging this translational divide [5]. Among them, patient-derived xenograft (PDX) models preserve the histological architecture and genetic landscape of the original tumors, whereas patient-derived organoids (PDOs) and organ-on-a-chip (OoC) systems further integrate three-dimensional organization, microenvironmental cues, and fluidic dynamics to better approximate human physiological conditions. These advanced models have been successfully applied to the preclinical validation of specific therapeutic targets, such as HER2 and CLDN18.2, and have contributed to the clinical translation and regulatory approval of therapies including trastuzumab and zolbetuximab, highlighting their potential in precision patient stratification and therapeutic response prediction [6].

Therefore, this review aims to systematically summarize the functional characterization of key gastric cancer–associated genes, findings from animal studies, and outcomes of clinical trials. Furthermore, we discuss the limitations of traditional animal models in translational research and explore how emerging platforms—such as PDX, PDOs, and OoC technologies-are reshaping the future of gastric cancer drug development and precision oncology.

## 2. Discovery of Gastric Cancer–Associated Genes

During the initiation and progression of gastric cancer, a series of critical genetic alterations accumulate, exerting profound effects on tumor development, progression, and clinical behavior. As early as 1991, Tamura, G. et al. and Yamada, Y. et al. independently reported a high frequency of *p53* (*TP53*) gene mutations in gastric cancer tissues, indicating that loss of tumor suppressor function plays a central role in gastric carcinogenesis [7,8]. Subsequently, in 1992, Nakatsuru, S. et al. investigated sporadic gastric cancers and identified somatic mutations in the *APC* gene in approximately 30% of cases, implicating the Wnt signaling pathway as a key contributor to intestinal-type gastric cancer development [9].

In 1998, Guilford, P. et al. first elucidated the molecular basis of hereditary diffuse gastric cancer in a landmark study published in *Nature* [10]. Their findings demonstrated germline mutations in the *CDH1* gene—including point mutations and frameshift mutations in exons 7, 13, and 15—in multiple familial gastric cancer cases, highlighting the disruption of cell–cell adhesion as a fundamental pathogenic mechanism in diffuse-type gastric cancer [10]. In addition, Oh, H.A. et al. reported that c-MYC protein expression was upregulated in approximately 64% of gastric cancer specimens and was significantly correlated with TNM stage, suggesting that c-MYC promotes tumor progression [11].

With the advent of molecular targeted research, Tanner, M. et al. reported that *HER2/neu* gene amplification was detected in 12.2% of gastric cancers and 24.0% of gastroesophageal junction adenocarcinomas, providing a critical foundation for the subsequent development of HER2-targeted therapies in gastric cancer [12]. Furthermore, in 2010, Ling, Z.Q. et al. demonstrated that mismatch repair (MMR) genes, particularly MLH1, frequently undergo promoter hypermethylation during gastric cancer development, leading to gene silencing and the emergence of microsatellite instability (MSI). These molecular alterations have since become key determinants in gastric cancer molecular classification [13].

With the maturation of next-generation sequencing technologies, Wang, K. et al. performed whole-exome sequencing on 22 gastric cancer samples and identified frequent inactivating mutations or protein loss of ARID1A in 83% of MSI-type, 73% of Epstein–Barr virus (EBV)-associated, and 11% of microsatellite-stable (MSS) gastric cancers, underscoring the role of chromatin remodeling dysregulation in gastric tumorigenesis [14]. Consistently, The Cancer Genome Atlas (TCGA) project identified recurrent PIK3CA mutations, which encode the p110α catalytic subunit of phosphatidylinositol 3-kinase (PI3K), in EBV-positive gastric cancers, further underscoring the critical role of PI3K/AKT signaling in the pathogenesis of this molecular subtype [15]. Moreover, Kakiuchi, M. et al. conducted exome sequencing of diffuse-type gastric cancers and identified nonsynonymous RHOA mutations in approximately 25.3% of cases. These mutations were shown to exert gain-of-function effects that promote tumorigenesis, thereby enriching the current understanding of the molecular mechanisms driving diffuse-type gastric cancer [16].

## 3. Functional Classification of Gastric Cancer-Associated Genes

**Figure 2:**
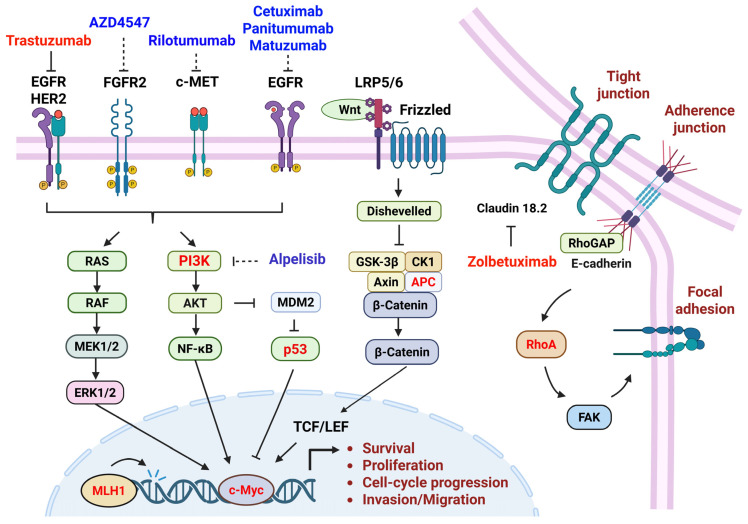
Overview of major signaling pathways involved in gastric cancer, including EGFR/HER2, FGFR2, c-MET, and downstream PI3K/AKT/mTOR and MAPK signaling, as well as alterations in WNT signaling, tight junctions, adherens junctions, and focal adhesions, which collectively contribute to tumor growth, invasion, and metastasis. Representative targeted therapies evaluated in gastric cancer are shown, including trastuzumab, AZD4547, cetuximab, panitumumab, matuzumab, rilotumumab, alpelisib, and zolbetuximab. While multiple pathways have demonstrated preclinical relevance, only HER2 and CLDN18.2 targeting have translated into consistent clinical benefit, highlighting the importance of oncogenic dependency and biomarker-driven patient selection. This figure was created in BioRender.

Gastric cancer does not arise from a single genetic alteration but rather results from the cumulative dysregulation of multiple genes with distinct biological functions. Based on their roles in cellular biology, gastric cancer–associated genes can be broadly categorized into four major groups (Table 1 and Figure 2): (i) oncogenes, (ii) tumor suppressor genes, (iii) genes involved in DNA repair and genomic stability, and (iv) genes regulating cellular structure, adhesion, and cytoskeletal dynamics. Although these genes participate in different functional pathways, they collectively influence cell proliferation, survival, genomic integrity, and invasive behavior, thereby driving gastric tumor initiation and progression.

### 3.1. Oncogenes

Oncogenes normally regulate cell growth and signal transduction in physiological conditions; however, upon mutation, amplification, or overexpression, they become constitutively activated, allowing cells to escape normal growth control. In gastric cancer, *HER2*, *PIK3CA*, and *c-MYC* are among the most representative oncogenes.

HER2 encodes a receptor tyrosine kinase whose gene amplification leads to persistent activation of the downstream MAPK and PI3K/AKT signaling pathways, thereby promoting tumor cell proliferation and resistance to apoptosis [17]. Activating mutations in PIK3CA directly stimulate the PI3K/AKT/mTOR signaling cascade and are particularly prevalent in EBV-associated gastric cancer [18,19]. c-MYC, a transcription factor, drives rapid tumor cell proliferation by regulating genes involved in cell cycle progression, metabolism, and ribosome biogenesis. Its overexpression has been strongly associated with aggressive tumor behavior and poor prognosis in gastric cancer [20,21].

HER2 amplification induces ligand-independent receptor dimerization and persistent activation of MAPK and PI3K/AKT signaling, whereas PIK3CA mutations constitutively activate the PI3K/AKT/mTOR axis. These signaling dependencies define critical nodes within proliferative and survival pathways in gastric cancer [22,23,24].

### 3.2. Tumor Suppressor Genes

Tumor suppressor genes function to restrain cell proliferation, facilitate DNA damage repair, and induce apoptosis. Loss of their function predisposes cells to mutation accumulation and malignant transformation. *TP53*, *APC*, and *ARID1A* are key tumor suppressor genes frequently altered in gastric cancer.

TP53, often referred to as the “guardian of the genome”, plays a central role in maintaining genomic integrity. Mutations in TP53 impair cellular responses to DNA damage, leading to chromosomal instability and tumor progression [25,26]. APC is a negative regulator of the Wnt/β-catenin signaling pathway, and its inactivation results in nuclear accumulation of β-catenin, thereby promoting tumorigenesis, particularly in intestinal-type gastric cancer [27,28]. ARID1A, a core component of the SWI/SNF chromatin remodeling complex, regulates chromatin accessibility and gene transcription. Loss of ARID1A function disrupts epigenetic regulation and is strongly associated with MSI and EBV-positive gastric cancer [29,30].

The loss of TP53, APC, and ARID1A collectively disrupts genome surveillance, Wnt signaling control, and chromatin remodeling, reshaping transcriptional and DNA repair networks in gastric tumorigenesis [31,32].

### 3.3. Genes Involved in DNA Repair and Genomic Stability

Genomic stability is fundamental to the maintenance of normal cellular function. MMR genes, such as *MLH1*, play a critical role in correcting DNA replication errors. Epigenetic silencing of *MLH1* through promoter hypermethylation leads to MSI, markedly increasing the mutation rate and thereby promoting gastric carcinogenesis. MSI-high gastric cancers are characterized by a high mutational burden and exhibit distinct molecular and clinical features [33,34]. Epigenetic silencing of MLH1 via promoter hypermethylation leads to microsatellite instability, increased mutation burden, and contributes to chemoresistance, emphasizing its relevance for treatment response prediction [35,36].

Epigenetic silencing of MLH1 impairs mismatch repair, resulting in microsatellite instability, increased insertion–deletion mutations, and elevated neoantigen load. This genomic instability alters tumor–immune interactions and defines a distinct molecular subtype of gastric cancer [37,38].

### 3.4. Genes Regulating Cellular Structure, Adhesion, and the Cytoskeleton

Recent studies have highlighted the importance of genes involved in cellular architecture and morphology, particularly in diffuse-type gastric cancer. Among these, *CDH1* and *RHOA* are representative examples.

*CDH1*, which encodes E-cadherin, is essential for maintaining cell–cell adhesion and epithelial integrity. Mutations in CDH1 disrupt intercellular junctions, facilitating tumor cell detachment, invasion, and dissemination [39,40]. RHOA regulates cytoskeletal organization and cell motility. Gain-of-function mutations in RHOA alter cellular morphology and mechanical properties, thereby promoting the infiltrative growth pattern characteristic of diffuse-type gastric cancer [41,42].

*CDH1* mutations disrupt E-cadherin–mediated adhesion and epithelial polarity, facilitating invasion and β-catenin–dependent transcription. Gain-of-function RHOA mutations alter actin dynamics and ROCK signaling, promoting the infiltrative growth characteristic of diffuse-type gastric cancer [43,44].

### 3.5. Epigenetic Alterations and Chemoresistance in Gastric Cancer

Beyond genetic mutations, epigenetic dysregulation—including DNA methylation, histone modifications, and non-coding RNA-mediated regulation—plays a central role in gastric cancer progression and therapeutic response. These alterations can modify chromatin structure and gene expression without changing the underlying DNA sequence, contributing to tumor heterogeneity and adaptive resistance to therapies, including cytotoxic chemotherapy and targeted agents [45].

DNA methyltransferases, histone deacetylases, and histone methyltransferases regulate gene expression programs controlling apoptosis, stemness, DNA repair, and drug metabolism. Aberrant activation or repression of these enzymes can reprogram oncogenic signaling pathways such as PI3K/AKT, Wnt/β-catenin, and EMT-associated networks, thereby reducing chemosensitivity and promoting acquired resistance to platinum-based agents, fluoropyrimidines, and targeted therapies [46].

MicroRNAs further contribute to epigenetic regulation by post-transcriptionally modulating oncogenes and tumor suppressor genes. Through coordinated regulation of multiple targets involved in apoptosis signaling, EMT, and drug efflux transporters, dysregulated microRNAs may enhance phenotypic plasticity and directly mediate chemoresistance in gastric cancer [47].

Collectively, these epigenetic alterations not only shape tumor evolution but also function as dynamic regulators of therapeutic response. By modulating DNA damage repair capacity, apoptotic thresholds, cancer stemness, and tumor microenvironment interactions, epigenetic mechanisms create a permissive landscape for chemotherapy resistance. This integrated molecular framework highlights critical nodes within MAPK, PI3K/AKT/mTOR, Wnt/β-catenin, DNA repair, and chromatin remodeling pathways that may be therapeutically exploitable. A mechanistic understanding of these interconnected networks provides the biological foundation for rational pathway-directed strategies and epigenetic combination approaches aimed at overcoming chemoresistance in gastric cancer.

## 4. Animal Models for Gastric Cancer–Associated Genes

Animal models have played a central role in elucidating the functional significance of gastric cancer-associated genes and in evaluating therapeutic vulnerabilities. Unlike certain cancers in which single-driver GEMMs faithfully recapitulate tumorigenesis, gastric cancer GEMMs are less frequently driven by individual gene alterations. Instead, most established models rely on combinatorial genetic perturbations to mimic the molecular subtypes and multistep evolution observed in human gastric cancer. These compound models integrate alterations in tumor suppressor genes, oncogenes, and signaling pathways, thereby more accurately reflecting disease complexity (Table 2).

### 4.1. CDH1

*Cdh1* (*E-cadherin*) is one of the most representative tumor suppressor genes in diffuse-type gastric cancer. Using gastric epithelium-specific GEMMs and complementary organoid systems, Zou, G. et al. systematically dissected the functional role of *Cdh1* loss in gastric tumorigenesis [48]. Their study demonstrated that loss of *Cdh1* alone is insufficient to immediately induce invasive tumors, but when combined with oncogenic Kras^G12D^ activation or *Trp53* deletion, it markedly accelerates the development and progression of diffuse-type gastric cancer.

Mechanistically, *Cdh1* loss not only disrupts epithelial cell–cell adhesion but also induces epigenetic reprogramming, particularly through EZH2-mediated chromatin regulation, leading to the activation of tumor-promoting gene networks. In addition, tumors arising in this model exhibit an immunosuppressive tumor microenvironment, indicating that CDH1 deficiency contributes to immune evasion. Collectively, these findings establish Cdh1 loss as a driver event in diffuse-type gastric cancer rather than a passive consequence of tumor progression.

### 4.2. CLDN18

Claudin-18.2 (CLDN18.2), a gastric lineage-restricted isoform of CLDN18, is a tight junction protein essential for maintaining gastric mucosal barrier integrity. Using *Cldn18* knockout mice, Hagen, S.J. et al. provided the first direct in vivo evidence that loss of *Cldn18* alone is sufficient to initiate gastric tumor-associated pathology [49]. Aging *Cldn18*^−/−^ mice developed progressive disruption of the gastric epithelial barrier, chronic inflammation, glandular hyperplasia, and preneoplastic lesions, ultimately giving rise to adenocarcinoma-like changes.

Mechanistic analyses revealed that *Cldn18* deficiency impairs gastric acid barrier function, triggering inflammatory responses and proliferative signaling cascades that promote tumorigenesis. Subsequent studies further demonstrated that during tumor progression, a distinct population of Lgr5^+^ stem cell-like tumor cells emerges within gastric tumors [50]. These cells exhibit high proliferative and self-renewal capacity and are thought to drive tumor maintenance and progression. Together, these animal studies firmly establish CLDN18 as a gastric tumor suppressor, whose loss contributes to both tumor initiation and malignant progression.

### 4.3. ARID1A and PIK3CA

Animal studies have shown that conditional deletion of *Arid1a* in gastric epithelial cells results only in mild mucosal hyperplasia and inflammation. However, when *Arid1a* loss is combined with activating mutations in PIK3CA, gastric tumorigenesis is dramatically enhanced [51]. This genetic cooperation leads to pronounced mucosal hyperplasia, tumor formation, and disease progression.

Analysis of the tumor microenvironment revealed that ARID1A deficiency reshapes immune composition, inducing a type 2 immune-dominant microenvironment characterized by T helper 2 cells, M2 macrophages, and elevated IL-4/IL-13 signaling. Mechanistically, ARID1A loss promotes epigenetic dysregulation and activation of PI3K/AKT signaling, thereby enhancing the expression of immunosuppressive cytokines. These findings demonstrate that ARID1A mutations not only directly drive gastric tumorigenesis but also accelerate disease progression by orchestrating a protumorigenic immune landscape.

### 4.4. MYC and p53

Recent studies employing somatic engineering-based mouse models have systematically examined the cooperative roles of MYC overexpression and p53 loss (*Trp53*^−/−^) in gastric cancer development and metastasis. In these models, Leibold, J. et al. induced MYC activation alongside *Trp53* inactivation in gastric epithelial cells using viral delivery or genome-editing approaches [52]. This genetic combination efficiently drove the formation of highly invasive gastric tumors and significantly increased the incidence of distant metastases.

Pathological and molecular analyses revealed that MYC enhances tumor cell proliferation and metabolic reprogramming, while p53 loss compromises DNA damage checkpoints and apoptotic responses, resulting in strong oncogenic synergy. Notably, these tumors exhibited genotype-specific metastatic behaviors, including increased hematogenous dissemination and enhanced adaptability to diverse microenvironments. Overall, this model demonstrates that MYC and p53 co-alterations not only promote gastric cancer initiation but also critically determine tumor invasiveness and metastatic potential.

### 4.5. RHOA

Unlike classical tumor suppressor mutations, *RhoA* mutations in diffuse-type gastric cancer function are predominantly gain-of-function alterations. Haisheng Zhang, H. et al. generated xenograft mouse models using gastric cancer cells harboring DGC-specific RHOA mutations (e.g., Y42C and G17E) [53]. Compared with wild-type RHOA, mutant RHOA significantly enhanced tumor growth and survival in vivo.

Mechanistic studies revealed that these RHOA mutations aberrantly activate focal adhesion kinase (FAK) signaling, strengthening cell–extracellular matrix adhesion and mechanotransduction. As a result, tumor cells become highly dependent on FAK signaling for survival. Importantly, pharmacological inhibition of FAK selectively suppressed the growth of *RHOA*-mutant tumors in vivo. These findings establish RHOA gain-of-function mutations as oncogenic drivers in diffuse gastric cancer and highlight the RHOA–FAK axis as a therapeutically actionable vulnerability.

## 5. Translational Progress from Animal Models to Clinical Application in Gastric Cancer

Despite the rapid expansion of genomic knowledge in gastric cancer, only a limited number of molecular targets have successfully progressed from preclinical validation to effective clinical application. While numerous oncogenic alterations demonstrate compelling tumor-suppressive effects in animal models, many fail to translate into meaningful clinical benefit, underscoring the complexity of gastric cancer biology and its pronounced molecular heterogeneity. To date, HER2-directed therapy remains the most successful paradigm, representing a rare example in which robust preclinical evidence, biomarker-driven patient selection, and well-designed clinical trials converged to establish a new standard of care. Other targets, including the PI3K pathway, MET, and FGFR2, have shown variable and often context-dependent efficacy and remain under active clinical investigation. A summary of representative targeted therapies and their translational status is provided in Table 3.

### 5.1. HER2 (ERBB2)

In contrast to tumor-initiating alterations such as CDH1 or ARID1A loss, HER2 amplification primarily functions as a tumor maintenance driver in gastric cancer. Consequently, HER2 has not been extensively modeled in gastric-specific GEMMs for tumor initiation. Instead, its oncogenic dependency has been robustly validated using xenograft and PDX models, which provided the critical preclinical rationale for the successful clinical development of trastuzumab in HER2-positive gastric cancer.

Multiple preclinical studies have demonstrated that trastuzumab monotherapy significantly suppresses tumor growth in HER2-amplified gastric cancer cell line–derived xenografts and PDX models, with antitumor efficacy closely correlating with HER2 copy number and protein expression levels [54]. Subsequent studies further showed that combining trastuzumab with conventional chemotherapy produced additive or synergistic effects, resulting in more durable tumor control [55]. In addition, the antibody–drug conjugate T-DM1 (trastuzumab emtansine) exhibited enhanced antitumor activity in HER2-positive gastric cancer xenografts, confirming that HER2-mediated internalization can be exploited for efficient intracellular delivery of cytotoxic payloads [56].

Collectively, these animal studies established HER2 as a critical dependency for tumor maintenance and provided a strong preclinical foundation for the successful clinical translation of trastuzumab and its derivatives. Indeed, the HER2 pathway represents the most complete success story in gastric cancer, spanning molecular discovery, animal validation, biomarker-guided clinical trials, and regulatory approval [57]. Beyond trastuzumab, trastuzumab deruxtecan (Enhertu) has recently been approved as a standard therapy and expanded to multiple solid tumors (https://www.reuters.com/business/healthcare-pharmaceuticals/us-fda-approves-daiichi-astrazeneca-drug-treatment-solid-tumors-2024-04-05/, accessed on 17 December 2025), including gastric cancer, further reinforcing HER2 as a clinically actionable target.

### 5.2. PIK3CA/PI3K Pathway

Activating mutations in PIK3CA lead to aberrant activation of the PI3K/AKT/mTOR signaling cascade in gastric cancer. However, despite strong biological rationale, PI3K-targeted therapies have not yet received Food and Drug Administration (FDA) approval for gastric cancer. Early-phase clinical trials, such as NCT01613950 (https://clinicaltrials.gov/study/NCT01613950, accessed on 18 December 2025), primarily focused on evaluating the safety, tolerability, and preliminary efficacy of the PI3Kα-specific inhibitor alpelisib (BYL719) in patients with PIK3CA-altered gastric cancer, including combination strategies with HSP90 inhibitors. Another ongoing trial (NCT04526470) (https://clinicaltrials.gov/study/NCT04526470, accessed on 18 December 2025) is investigating alpelisib in combination with chemotherapy to enhance therapeutic benefit in molecularly selected patients.

Although these studies have not yet resulted in regulatory approval, early data suggest that PI3K pathway inhibition may provide clinical benefit in carefully selected molecular subgroups, with acceptable tolerability and increasingly refined dosing strategies. Importantly, the successful approval of alpelisib in PIK3CA-mutant HR^+^/HER2^−^ breast cancer highlights the potential of this pathway when precise patient selection is applied, suggesting that PI3K-targeted therapy in gastric cancer may still be feasible under optimized biomarker-driven strategies.

### 5.3. MET and FGFR2

Aberrant activation or amplification of MET (hepatocyte growth factor receptor) occurs in a subset of gastric cancers and has been associated with aggressive disease and therapeutic resistance. Several MET inhibitors have been evaluated in early- to mid-phase clinical trials. For example, AMG 337 was assessed in NCT02344810 to determine its safety and efficacy in MET-amplified gastric cancer, both as monotherapy and in combination with chemotherapy. Additional trials, such as NCT04923932, are evaluating savolitinib as a single agent, while NCT05620628 explores combination therapy with the immune checkpoint inhibitor durvalumab. These studies typically assess the objective response rate (ORR), progression-free survival (PFS), and safety, aiming to identify molecularly defined populations most likely to benefit. Although MET inhibitors have demonstrated antitumor activity in select patients, they have not yet established a new standard of care.

The FGFR2 pathway represents another promising therapeutic target in gastric cancer. Early-phase studies have evaluated FGFR2-targeted agents in molecularly defined populations. For example, a phase II study of futibatinib (TAS-120) in patients with FGFR2-amplified gastric or gastroesophageal junction cancer demonstrated modest antitumor activity with an objective response rate of 17.9% and a manageable safety profile, providing rationale for further investigation [58]. In addition, infigratinib has been assessed in a phase II trial (NCT05019794) in advanced FGFR2-altered gastric cancer, demonstrating an approximate 25% ORR and high disease control rate, including cases of significant tumor shrinkage [59].

Importantly, the FGFR2b-specific monoclonal antibody bemarituzumab has shown encouraging clinical outcomes: in the randomized phase II FIGHT trial (NCT03694522), bemarituzumab plus mFOLFOX6 improved progression-free and OS compared with placebo plus chemotherapy in FGFR2b-positive advanced gastric or gastroesophageal junction adenocarcinoma [60]. Moreover, recent top-line results from the phase III FORTITUDE-101 study demonstrate a significant OS benefit with bemarituzumab plus chemotherapy in patients with FGFR2b overexpression, further positioning FGFR2b-directed therapy as one of the most promising non-HER2 targeted strategies to date (https://www.amgen.com/newsroom/press-releases/2025/06/amgen-announces-positive-topline-phase-3-results-for-bemarituzumab-in-fibroblast-growth-factor-receptor-2b-fgfr2b-positive-firstline-gastric-cancer, accessed on 5 January 2026).

Among currently investigated molecular targets in gastric cancer, HER2 remains the only pathway with firmly established clinical benefit, while FGFR2b-targeted therapy appears to be the most promising emerging strategy. Other pathways, including PI3K and MET, continue to be explored within the framework of precision oncology and biomarker-guided clinical trial design.

## 6. Limited Translatability of Preclinical Models to Clinical Outcomes: Lessons from Failed Targeted Therapies in Gastric and Gastroesophageal Adenocarcinoma

Despite substantial advances in molecular characterization and preclinical drug development, the successful translation of animal study findings into effective clinical therapies remains a major challenge in oncology. A large-scale integrative analysis has estimated that only approximately 5% of therapeutic strategies demonstrating efficacy in animal models ultimately receive approval for human use [61]. This translational gap is particularly evident in gastric cancer research. Although a wide range of GEMMs, xenograft systems, and PDX have been employed for drug testing, intrinsic limitations—such as incomplete recapitulation of disease progression, differences in tumor microenvironment, and profound inter- and intratumoral heterogeneity—have contributed to the frequent failure of promising candidates in late-stage clinical trials [62]. To provide an overview of these translational failures, representative targeted therapies that demonstrated preclinical efficacy but failed to achieve clinical benefit in gastric or gastroesophageal adenocarcinoma are summarized in Table 4.

### 6.1. EGFR-Targeted Therapies (Matuzumab, Cetuximab, and Panitumumab)

Although EGFR overexpression is observed in a subset of gastric cancers and preclinical xenograft models demonstrated tumor growth inhibition upon EGFR blockade, these findings did not translate into clinical survival benefits in large randomized trials. The landmark EXPAND trial was a randomized, open-label phase III study evaluating the addition of cetuximab to capecitabine and cisplatin as first-line therapy in advanced gastric cancer. The results showed no improvement in PFS or OS, while treatment-related toxicity was increased [63]. Similarly, the UK-led REAL3 trial compared standard chemotherapy with or without panitumumab and failed to demonstrate an OS benefit, instead revealing a detrimental survival trend in the experimental arm [64]. Furthermore, the anti-EGFR monoclonal antibody matuzumab, when combined with epirubicin, cisplatin, and capecitabine (ECX) chemotherapy in a phase II trial, did not confer any clinical advantage [65]. Collectively, these studies indicate that EGFR expression or activation alone is insufficient to define therapeutic dependency in gastric cancer, highlighting the impact of molecular heterogeneity and compensatory signaling pathways on treatment resistance.

Taken together, the disappointing outcomes of EGFR-targeted strategies in gastric cancer highlight a critical disconnect between preclinical efficacy and clinical benefit. These experiences underscore that overexpression by IHC alone does not necessarily equate to oncogenic addiction or pathway dependency. Unlike tumor types such as colorectal or lung cancer—where EGFR-driven biology is more clearly defined—gastric cancer is characterized by substantial molecular heterogeneity, co-activation of parallel signaling networks, and dynamic pathway cross-talk. Consequently, therapeutic targeting based solely on receptor abundance without functional validation or robust genomic stratification may be insufficient. These lessons emphasize the importance of refined biomarker strategies, deeper biological context, and mechanism-based patient selection in future drug development efforts.

### 6.2. MET/HGF Pathway: Rilotumumab (RILOMET-1)

Aberrant activation of the MET pathway has been associated with aggressive tumor behavior and poor prognosis in a subset of gastric cancers, and preclinical models supported the inhibition of the HGF–MET axis as a therapeutic strategy. Based on this rationale, the RILOMET-1 trial was a large, randomized, double-blind phase III study evaluating the addition of the anti-HGF antibody rilotumumab to ECX chemotherapy in MET-positive advanced gastric cancer. Despite biomarker-based patient selection, the trial failed to demonstrate an OS benefit and even showed unfavorable outcomes in certain analyses, leading to early termination [66]. Subsequent evaluations suggested that immunohistochemistry (IHC)-based assessment of MET expression did not adequately identify tumors truly dependent on MET signaling, underscoring the limitations of non-functional biomarkers in translational oncology.

Collectively, the RILOMET-1 failure illustrates a central principle in precision oncology: pathway expression is not equivalent to pathway addiction. Without stringent enrichment for genomically amplified or functionally MET-dependent tumors, targeted inhibition may dilute potential benefit within a largely non-dependent population. This case underscores that biomarker precision—particularly the distinction between functional dependency and context-dependent signaling—is pivotal for overcoming translational failure in gastric cancer drug development.

### 6.3. FGFR2 and PI3K Pathways: Strong Preclinical Rationale but Modest Clinical Benefit

FGFR2 amplification and activation of the PI3K/AKT/mTOR pathway represent oncogenic drivers in subsets of gastric cancer, and inhibition of these pathways effectively suppresses tumor growth in cell-based and animal models. However, clinical outcomes have generally been modest. For FGFR2 inhibitors, partial tumor regression has been observed in FGFR2-amplified patients, but phase II trials failed to demonstrate consistent and durable survival benefits [67]. Similarly, PI3K pathway inhibitors in gastric cancer have largely remained in early-phase trials, showing disease stabilization rather than significant OS improvement, often limited by toxicity and intratumoral heterogeneity [68,69]. These findings suggest that single-pathway inhibition may be insufficient to overcome the molecular complexity of gastric cancer.

Collectively, these observations highlight a recurrent challenge in precision oncology: the presence of a genomic alteration does not necessarily guarantee sustained therapeutic dependency. Although high-level clonal FGFR2 amplification has been associated with meaningful responses in selected cases, such events are relatively rare and often coexist with substantial intra-tumoral heterogeneity. Moreover, adaptive feedback activation and parallel pathway signaling—particularly involving MAPK or alternative receptor tyrosine kinases—may attenuate the durability of single-agent inhibition. The limited efficacy observed with PI3K pathway inhibitors further reinforces the notion that targeting a single downstream node may be insufficient within a highly interconnected signaling network. These clinical experiences emphasize the need for more refined molecular stratification, functional validation of pathway addiction, and rational combination strategies to overcome adaptive resistance mechanisms in gastric cancer.

In summary, although multiple oncogenic pathways—including EGFR, MET, and FGFR2—have demonstrated compelling activity in preclinical gastric cancer models, most corresponding clinical trials failed to translate these findings into meaningful OS benefits. These recurring disappointments suggest that the central challenge is not merely target identification, but the accurate determination of true tumor dependency within a highly heterogeneous and dynamically adaptive disease. Overreliance on static biomarkers, coupled with preclinical systems that incompletely recapitulate human tumor–microenvironment interactions, has contributed to this translational gap.

Consequently, future therapeutic development must move beyond single-pathway inhibition and simplistic biomarker stratification toward integrated, functionally validated, and context-aware strategies. In this regard, the recent policy shift by the U.S. FDA in April 2025 to gradually phase out traditional animal testing requirements for certain drug classes and promote “human-relevant” New Approach Methodologies (NAMs)—including organoids, organ-on-chip platforms, and AI-based predictive modeling—reflects a broader recognition that next-generation translational platforms are essential for improving clinical predictability (https://www.fda.gov/news-events/press-announcements/fda-announces-plan-phase-out-animal-testing-requirement-monoclonal-antibodies-and-other-drugs, accessed on 18 December 2025). Together, these developments underscore a paradigm shift in gastric cancer drug development: from genomic identification to functional precision.

## 7. From Patient-Derived Models to Clinical Translation: PDX and Organoid-Based Approaches in Gastric Cancer

The repeated failure of promising targeted therapies to translate from animal models to clinical benefit has prompted the search for more human-relevant preclinical systems. In this context, PDX and PDOs have emerged as powerful platforms that more faithfully recapitulate the genetic heterogeneity, histopathological features, and drug response profiles of individual gastric tumors. Accumulating evidence indicates that drug sensitivity patterns observed in gastric cancer organoids correlate strongly with patient clinical responses, supporting their utility in personalized drug screening and resistance mechanism studies [70].

Despite these advantages, large-scale regulatory approvals in gastric cancer that rely directly on PDX or organoid data as pivotal evidence remain limited. To date, CLDN18.2-targeted therapy represents one of the few notable exceptions in which extensive validation using patient-derived models contributed meaningfully to successful clinical translation. CLDN18.2 is a tight junction protein selectively expressed in gastric mucosal epithelial cells and represents the gastric-specific isoform of the CLDN18 gene, whereas CLDN18.1 is predominantly expressed in lung epithelium. In normal tissue, CLDN18.2 is largely sequestered within intercellular junctions and is therefore poorly accessible to antibody-based therapies. During malignant transformation, however, loss of epithelial polarity and disruption of tight junction architecture lead to aberrant surface exposure of CLDN18.2 on tumor cells, rendering it an attractive and tumor-selective therapeutic target [71].

Zolbetuximab (IMAB362) is a humanized IgG1 monoclonal antibody that specifically binds CLDN18.2 and induces tumor cell death primarily through antibody-dependent cellular cytotoxicity [72]. Owing to the lineage-restricted expression of CLDN18.2 in gastric tissue and its minimal accessibility in normal organs, CLDN18.2-directed therapy offers a favorable therapeutic window and avoids off-target effects associated with the lung-specific CLDN18.1 isoform.

The clinical efficacy of zolbetuximab has been confirmed in two international, multicenter phase III trials: the SPOTLIGHT trial (zolbetuximab plus mFOLFOX6) [73] and the GLOW trial (zolbetuximab plus CAPOX) [74]. Both studies demonstrated significant improvements in PFS and OS compared with chemotherapy alone in patients with CLDN18.2–high advanced gastric or gastroesophageal junction adenocarcinoma, with an acceptable safety profile [74]. Based on these results, zolbetuximab received regulatory approval in multiple countries in 2024 for the treatment of selected CLDN18.2-positive gastric cancer patients.

Importantly, during its preclinical development, extensive use of PDX and patient-derived organoid models was instrumental in validating antibody binding, tumor dependency, and immune-mediated effector mechanisms. These studies provided strong translational confidence that CLDN18.2 represents a biologically relevant and therapeutically actionable target. As such, zolbetuximab stands as one of the rare examples in gastric cancer in which a therapy successfully completed the full trajectory from patient-derived preclinical models to clinical approval.

## 8. Comparative Lessons from Translational Successes and Failures: HER2 and CLDN18.2 Versus EGFR, MET, and Related Targets

In contrast to the repeated clinical failures observed with EGFR, MET, FGFR2, or PI3K pathway inhibition in gastric cancer, HER2 and CLDN18.2 represent two of the few targets that have successfully completed the full translational continuum from preclinical validation to regulatory approval. The critical distinctions between these successful and unsuccessful cases lie in tumor dependency, biomarker precision, and mechanistic clarity.

HER2 amplification constitutes a well-defined oncogenic driver event in gastric cancer, characterized by stable and reproducible gene amplification and protein overexpression. This dependency can be reliably assessed using standardized IHC and fluorescence in situ hybridization (FISH), enabling accurate patient selection. Preclinical studies consistently demonstrated strong trastuzumab dependency in HER2-overexpressing gastric cancer models, and the landmark ToGA trial confirmed that HER2-targeted therapy significantly improves OS when administered to rigorously selected HER2-positive patients [22].

CLDN18.2 exemplifies a distinct but equally successful translational paradigm. Its expression is highly gastric-specific, and in normal tissue, its localization within tight junctions limits antibody accessibility. Malignant transformation results in widespread surface exposure of CLDN18.2, creating an ideal target for antibody-mediated cytotoxicity [75,76]. Genetic deletion or functional loss of CLDN18 in mouse models leads to gastric mucosal barrier disruption and tumor progression, further supporting its biological relevance. Clinically, zolbetuximab trials have demonstrated significant improvements in both PFS and OS in patients with high CLDN18.2 expression [73,74].

By contrast, EGFR and MET are frequently overexpressed or activated in subsets of gastric cancer but often represent “passenger” or context-dependent alterations rather than true oncogenic drivers [63,64]. The lack of robust functional biomarkers for defining pathway dependency, combined with profound molecular heterogeneity and compensatory signaling networks, has limited the clinical efficacy of single-pathway inhibition strategies targeting these pathways (Figure 1).

Beyond individual trial outcomes, the distinguishing feature of HER2- and CLDN18.2-directed therapies appears to be the presence of biologically meaningful tumor dependency coupled with stable and spatially consistent target expression. In HER2-positive gastric cancer, gene amplification represents a clonal oncogenic driver event rather than a secondary or context-dependent alteration, enabling sustained pathway addiction and therapeutic vulnerability. Similarly, CLDN18.2 benefits from lineage-restricted and relatively homogeneous expression, as well as tumor-specific surface exposure following malignant transformation, facilitating effective antibody-mediated targeting.

In contrast, EGFR and MET alterations in gastric cancer are frequently heterogeneous, dynamically regulated, or embedded within redundant signaling networks, limiting true pathway dependency. Therefore, the critical determinant of clinical success may not be target expression per se, but whether the tumor exhibits functional and sustained reliance on that target, supported by robust and reproducible biomarker-driven stratification. Among these factors, true oncogenic dependency and stable target expression likely represent the most fundamental requirements, while precise patient stratification serves as the enabling translational tool. The key conceptual distinctions are summarized in Table 5.

## 9. Conclusions and Future Perspectives

Over the past decades, substantial progress has been made in dissecting the molecular underpinnings of gastric cancer, and targeted therapies such as HER2 and CLDN18.2 inhibitors have demonstrated that precision oncology can significantly improve patient outcomes. However, the high rate of clinical failure for many promising agents—despite robust efficacy in traditional in vitro and animal models—underscores a critical translational gap. This gap is driven by multiple factors, including the limitations of conventional animal models in capturing human tumor complexity, differences in tumor microenvironment and immune contexture, and the lack of highly predictive biomarkers for true oncogenic dependency. These challenges highlight the need for more human-relevant preclinical systems that can better inform clinical responsiveness and reduce attrition in late-stage trials.

In response to these limitations, patient-derived models such as organoids and PDXs have gained traction as complementary tools that maintain the genetic heterogeneity and, in many cases, drug response profiles of primary tumors. Gastric cancer organoids established from endoscopic biopsy specimens retain histopathological and molecular features of the original tumors, providing a platform for drug screening and mechanistic studies that more closely reflect human disease biology [77,78]. Despite these advancements, regulatory approvals based directly on organoid or PDX evidence are still rare, with the CLDN18.2-targeted therapy zolbetuximab being one of the few success stories where these models contributed critically to the translational pipeline [79,80].

To further improve translational success, future studies should integrate biomarker-guided trial designs and molecular stratification criteria. For example, patient selection based on validated functional biomarkers—such as HER2 amplification or CLDN18.2 surface expression—can enrich for populations truly dependent on the target pathway. Complementary preclinical approaches should prioritize model selection strategies that preserve tumor heterogeneity and recapitulate human microenvironmental context, such as using organoids, PDX, or hybrid organoid-on-chip platforms. Together, these approaches can inform more predictive dose schedules, combination strategies, and endpoint selection, ultimately bridging the gap between preclinical promise and clinical efficacy.

Advances in microphysiological systems, particularly OoC and organoid-on-chip technologies, promise to further reshape preclinical modeling by integrating human cells within microengineered environments capable of simulating key aspects of organ function, dynamic physiological flow, multicellular interactions, and mechanical forces that are not recapitulated in static cultures or animal models [81,82]. These systems have demonstrated utility in oncology research by more accurately capturing tumor–stroma interactions, vascularization processes, and therapeutic responses compared to traditional models, thereby enhancing human-relevant data generation for drug discovery and toxicity testing.

Importantly, specific gastric cancer molecular subtypes—such as EBV-positive and MSI-high tumors—exhibit distinct immunogenic profiles and treatment sensitivities. Failure to consider these subtypes in past clinical trials likely contributed to the modest efficacy observed for many targeted agents, as patients without true oncogenic dependency or with immune-inflamed tumors may not respond to single-pathway inhibition. Incorporating molecular stratification criteria, including MSI status, EBV positivity, and immune biomarkers (e.g., PD-L1, tumor mutational burden), into trial design—combined with patient-derived models that recapitulate both tumor genomics and microenvironmental context—offers a practical strategy to improve translational success and guide rational combination therapies.

Concurrently, regulatory landscapes are evolving to support these innovations. The FDA has explicitly embraced the use of New Approach Methodologies—including organoids, OoC platforms, computational models, and other human-based systems—to reduce reliance on animal testing, improve the predictive accuracy of preclinical testing, and accelerate drug development timelines [83]. The FDA’s 2025 “Roadmap to Reducing Animal Testing” outlines a strategic, phased plan to integrate New Approach Methodologies into regulatory decision making, with pilot programs allowing human-relevant data—including from OoC systems—to inform safety and efficacy evaluations traditionally supported by animal studies (https://www.fda.gov/files/newsroom/published/roadmap_to_reducing_animal_testing_in_preclinical_safety_studies.pdf, accessed on 18 December 2025). Such initiatives reflect a paradigm shift that positions human-derived models at the forefront of translational oncology.

Looking forward, the convergence of advanced in vitro platforms, AI-based modeling, and harmonized regulatory frameworks will be essential for realizing a new era of precision preclinical research in gastric cancer. Hybrid systems that combine organoids or OoC data with AI-driven predictive analytics hold particular promise for addressing tumor heterogeneity and therapeutic resistance, potentially improving the success rate of clinical translation while reducing ethical and practical burdens associated with animal studies [84]. Nevertheless, challenges remain in standardizing model protocols, ensuring reproducibility, and establishing robust validation pathways across diverse therapeutic contexts.

## Figures and Tables

**Table 1:** Functional classification of gastric cancer–associated genes.

Functional Category	Gene	Primary Mechanism of Action	Biological Impact in Gastric Cancer
Oncogenes	*HER2*	RTK activation; MAPK/PI3K signaling	Proliferation, anti-apoptosis
	*PIK3CA*	PI3K/AKT pathway activation	Survival, metabolic reprogramming
	*c-MYC*	Transcriptional activation; cell cycle control	Malignant progression
Tumor suppressors	*TP53*	DNA damage checkpoint; apoptosis	Genomic instability
	*APC*	Inhibition of Wnt/β-catenin signaling	Intestinal-type tumorigenesis
	*ARID1A*	Chromatin remodeling	Epigenetic dysregulation
DNA repair-related	*MLH1*	Mismatch repair	Microsatellite instability
Structural genes	*CDH1*	Cell–cell adhesion	Invasion and metastasis
	*RHOA*	Cytoskeletal regulation	Diffuse-type gastric cancer

RTK, Receptor tyrosine kinase; MAPK, Mitogen-activated protein kinase; PI3K, Phosphoinositide 3-kinase.

**Table 2:** Summary of animal models for gastric cancer-associated genes.

Gene(s)	Animal Model	Model System	Key Findings
*Cdh1*/*Trp53*/*Kras*	*Cdh1* KO + *Trp53* KO + *Kras*^G12D^ (C57BL/6)	GEMMs	*CDH1* loss accelerates diffuse-type gastric cancer, promotes immune evasion, and cooperates with EZH2-driven oncogenic pathways
*Cldn18*	*Cldn18*^−/−^ mice	Germline knockout	Spontaneous gastritis, glandular hyperplasia, and preneoplastic lesions; CLDN18 functions as a gastric tumor suppressor
	Tumor progression models	Murine gastric cancer models	Lgr5^+^ stem cell–like tumor populations sustain advanced gastric cancer
*Arid1a*/*Pik3ca*	*Arid1a*^flox^ + *Pik3ca*^H1047R^	Stomach-specific GEMMs	*ARID1A* loss alone causes hyperplasia; combination with *PIK3CA* activation drives tumorigenesis
*Myc*/*Trp53*	*Myc*-driven/*Trp53*^−/−^	Electroporation-based GEMMs	Progression from adenoma to invasive carcinoma with high metastatic potential
*RhoA*	Mutant *RhoA* xenografts	Immunodeficient mice	Gain-of-function *RHOA* mutations enhance tumor growth and induce FAK-dependent oncogenic addiction

GEMMs, genetically engineered mouse models.

**Table 3:** Representative targeted therapies in gastric cancer: from molecular targeting to clinical development.

Molecular Target	Representative Agent	Patient Selection	Trial Phase	Primary Endpoints	Current Status/Key Outcomes
HER2 (ERBB2)	Trastuzumab	HER2 amplification/IHC 3^+^	Phase III	OS, PFS, ORR	Significantly improved OS; established as standard therapy (ToGA trial)
	T-DM1 (trastuzumab emtansine)	HER2-positive GC	Phase I/II	ORR, safety	Demonstrated antitumor activity but did not surpass trastuzumab
	Pertuzumab + trastuzumab	HER2-positive GC	Phase II/III	OS, PFS	Combination explored; limited incremental benefit
PIK3CA/PI3K pathway	Alpelisib + paclitaxel	PIK3CA mutation/pathway activation	Phase I/II	ORR, PFS, safety	Acceptable safety; efficacy enriched in molecularly selected subgroups
	Capivasertib + paclitaxel	PIK3CA mutation/amplification	Phase II	ORR	No definitive breakthrough to date
MET (c-MET)	AMG 337	MET amplification	Phase I/II	ORR, safety	Partial responses in small cohorts
	Savolitinib	MET amplification/overexpression	Phase II	ORR, PFS	Limited single-agent activity
	Savolitinib + durvalumab	MET-positive GC	Phase I/II	ORR, safety	Combination strategies under evaluation
FGFR2	Futibatinib (TAS-120)	FGFR2 amplification/fusion	Phase I/II	ORR, safety	Early signs of activity
	Infigratinib	FGFR2-altered GC/GEJ	Phase II	ORR	Tumor shrinkage in selected cases
	Bemarituzumab (FGFR2b mAb)	FGFR2b overexpression	Phase II/III	OS, PFS	Survival benefit with chemotherapy; most promising FGFR2 strategy

IHC, immunohistochemistry; GC, gastric cancer; OS, overall survival; PFS, progression-free; ORR, objective response rate.

**Table 4:** Representative examples of targeted therapies in gastric or gastroesophageal adenocarcinoma that demonstrated preclinical efficacy but failed to achieve clinical benefit.

Target Gene/Pathway	Representative Drug/Trial	Preclinical Evidence	Key Clinical Trial (Reference)	Clinical Outcome	Major Cause of Translational Failure
EGFR	Cetuximab	Suppressed gastric cancer xenograft growth	EXPAND [63]	No improvement in OS or PFS	Lack of EGFR dependency; compensatory signaling
	Panitumumab	Inhibited cell proliferation	REAL3 [64]	Significantly reduced OS	Inappropriate patient selection; toxicity
	Matuzumab	Xenograft tumor regression	Phase II [65]	No significant clinical benefit	EGFR not a dominant driver
MET/HGF	Rilotumumab	Inhibited MET-driven tumor growth	RILOMET-1 [66]	No OS benefit; trial terminated early	MET IHC not a functional biomarker
FGFR2	AZD4547, others	High sensitivity in FGFR2-amplified models	[67]	Partial responses; OS not confirmed	Intratumoral heterogeneity

OS, overall survival; PFS, progression-free; IHC, immunohistochemistry.

**Table 5:** Comparative features underlying translational success and failure of targeted therapies in gastric cancer.

Factor	HER2	CLDN18.2	EGFR/MET
**True driver dependency**	Yes (oncogenic addiction in amplified tumors)	Not classical oncogenic driver; lineage-restricted target	Amplification-driven in subset; otherwise context-dependent
**Stable expression**	Gene amplification (stable)	Lineage-restricted, relatively stable	Heterogeneous, dynamic
**Biomarker precision**	IHC + FISH standardized	IHC threshold defined	Variable; no universally standardized algorithm
**Intratumoral uniformity**	Moderate; often dominant clone but heterogeneous	Often diffuse	Frequently patchy
**Resistance bypass**	Emergent bypass via parallel RTKs	Non–signaling structural target; immune-mediated mechanism	Extensive pathway redundancy

IHC, immunohistochemistry; FISH, fluorescence in situ hybridization; RTK; receptor tyrosine kinase.

## Data Availability

No new data were created or analyzed in this study.

## Abbreviations

The following abbreviations are used in this manuscript:

APC	Adenomatous polyposis coli
GEMMS	Genetically engineered mouse models
EGFR	Epidermal growth factor receptor
FGFR2	Fibroblast growth factor receptor 2
OS	Overall survival
CLDN18.2	Claudin-18.2
PDX	Patient-derived xenograft
PDOs	Patient-derived organoids
OoC	Organ-on-a-chip
MMR	Mismatch repair
MSI	Microsatellite instability
MSS	Microsatellite-stable
PI3K	Phosphatidylinositol 3-kinase
EBV	Epstein–Barr virus
RTK	Receptor tyrosine kinase
MAPK	Mitogen-activated protein kinase
FAK	Focal adhesion kinase
IHC	Immunohistochemistry
PFS	Progression-free
OSS	Objective response rate
FDA	Food and Drug Administration
ECX	Epirubicin, cisplatin and capecitabine
NAMs	New Approach Methodologies
AI	Artificial intelligence
